# Subshell Stability in Superatomic Clusters and the Formation of Stable Magnetic Motifs

**DOI:** 10.1021/acs.jpca.5c07616

**Published:** 2026-03-09

**Authors:** Deepak Kumar, Arthur C. Reber, Shiv N. Khanna

**Affiliations:** Physics Department, 6889 Virginia Commonwealth University, Richmond, Virginia 23284-2000, United States

## Abstract

It is now well established that the quantum states in symmetric clusters are grouped into shells and that clusters with filled shells exhibit enhanced stability. While the stability with filled shells is established, the corresponding stability associated with half-filled shells and its role in properties is largely unexplored. In this work, we first examine the stability due to half-filled shells by considering a variety of clusters and show that such fillings indeed enhance the energetic stability. We then demonstrate that such a possibility enables the formation of magnetic species via stable subshells belonging to different quantum numbers. The formation of stable magnetic units with filled subshells opens the door to creating magnetic nanoassemblies with tunable coupling and magnetic anisotropy.

## Graphical abstract

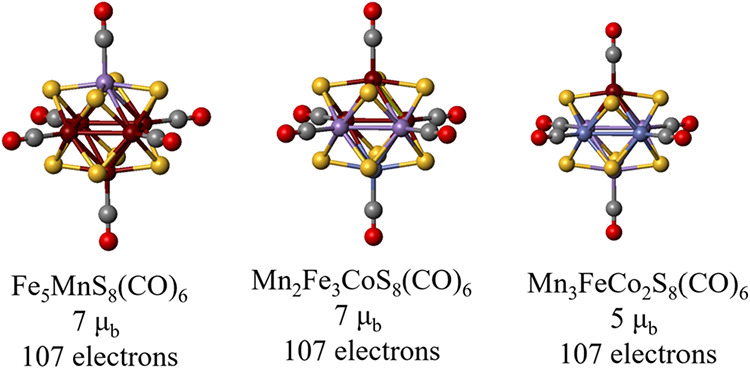

## I Introduction

One of the fundamental discoveries in atomic clusters and nanostructures is the grouping of quantum states into shells similar to atoms or nuclei.^1–4^ The existence of electronic shells has now been demonstrated in a variety of symmetric clusters including simple, semiconducting, metal-chalcogenide and ligated noble metal clusters.^5,6^ The shell structure determines the physical, chemical, electronic and magnetic behaviors to the extent that each electron and each atom can change the cluster properties including stability.^7–10^ For example, clusters with filled shells separated by large gaps from unfilled shells are found to exhibit enhanced energetic stability and chemical inertness. These findings were first seen in simple metal clusters (Na*_n_*, K*_n_* etc.), where measurements of abundance spectra, ionization energies, electron affinities, polarizability etc. indicated that electron counts of 2, 8, 18, 20, 34, 40, 70.. (called magic numbers^11^) lead to stable species as seen through peaks in mass spectrum, high ionization energies, lower electron affinities and polarizability.^1,12–16^ These electron counts further lead to chemical inertness as was first demonstrated through reactivity of anionic Al*_n_* ^–^ clusters with oxygen.^8,9^ Experiments showed that while most clusters reacted with oxygen as in case of bulk aluminum, selected clusters including Al_13_^–^, Al_23_^–^,.. were resistant to reactivity.^8,9^ Noting that Al is trivalent, these sizes correspond to electron counts of 40, 70.. indicating that along with energetic stability, the magic sizes also lead to reduced reactivity. Theoretical investigations showed that the above magic numbers could be rationalized as derived from grouping of quantum states in a confined nearly free electron gas (CNFEG) where the quantum states order as 1S, 1P, 1D, 2S, 1F, 2P.. The clusters with filled electronic shells and large gaps between the highest occupied molecular orbital (HOMO) and lowest unoccupied molecular orbital (LUMO) are particularly stable and relatively inert, accounting for the magic numbers observed in experiments. These patterns are also found in bimetallic clusters such as aluminum–magnesium, silicon–lithium.^17–19^ Further studies in other clusters and more notably, ligated metal chalcogenide clusters marked by covalent bonds showed that the conceptual basis of magic sizes is not hostage to simple metal clusters.^20–26^ Detailed theoretical studies in octahedral transition metal chalcogenide clusters TM_6_E_8_L_6_ (TM: transition metal, E: Chalcogen atoms, L:Ligand) showed that they also have grouping of quantum shells that attain filled shell electronic configurations when they have 96, 100, or 114 valence electrons.^20–22^ These observations have led to a more modern conceptual basis of “superatoms” defined as clusters whose chemical, electronic, magnetic and chemical properties are dominated by their closeness to zerovalent state, just like atoms in the periodic table.^27–34^ The superatoms form a third dimension to the periodic table.

While the filling of shells by pairs of electrons leads to stable and inert species, an area of tremendous interest is the magnetic properties. Magnetism, by its very nature, requires unpaired electron balance to create local moments.^35,36^ Since the cluster stability requires filled electronic shells, it raises a fundamental dichotomy if one can attain both stability and magnetism in a given cluster.^37^ This is important for creating magnetic materials using stable magnetic clusters as building blocks. One way to attain this objective is to somehow explore if one could separately stabilize shells of up and down spin electrons with differential subshell filling. Such a possibility would create stable magnetic species via different filled subshells for spin up and down electrons.

The purpose of the paper is to first demonstrate that the stability arising from filled shells can be extended to subshells namely clusters with filled subshells also exhibit enhanced stability. As we will show, a subshell filling can arise from the gain in exchange energy via Hund’s coupling as in case of atoms.^35^ While the filled subshells provide stability, as we will show, loss of chemical symmetry or structural distortions can destroy this stability. This is because finite clusters with unlike atoms can either undergo structural distortions or broaden electronic shells that can destroy degeneracy of electronic shells. The stability of clusters then depends on a competition between Jahn–Teller distortion and exchange energy where a cluster can acquire stability either via structural distortion that breaks the spin symmetry or by keeping spatial symmetry and filling subshells by transferring minority electrons to complete majority subshell.^36,37^ In metallic clusters where the geometrical rearrangements require less energy, the Jahn–Teller wins leading to nonmagnetic ground states. However, for clusters with covalent bonds that offer rigidity, as we will show, subshell filling can be a dominant stabilizer particularly for homoatomic clusters. In fact, we show that this subshell stability allows for an alternate formulation where one can create stable magnetic motifs by filling majority and minority subshells belonging to different quantum numbers. Here, the magnetic moment is due to subshell occupations that offer electronic stability along with a stable magnetic moment. In the following, we outline the conditions necessary to form magnetic species and show the experimental evidence for their existence.

## II Theoretical Methods

The investigations used first-principles density functional theory using the Amsterdam Density Functional (ADF) program.^38^ The Perdew, Burke, and Ernzerhof (PBE) exchange-correlation functional was used.^39^ The basis set was the TZ2P basis with a large frozen electron core. The local minimum for each structure was found using the quasi-Newton method with no symmetry restriction and the lowest energy structures were ascertained. Relativistic effects were incorporated using the zero-order regular approximation (ZORA).^40,41^

## III Hund’s Rule vs Jahn–Teller in Small Metallic Clusters: Single Sub-Shell Closing

In the above, we have outlined how the electron counts lead to stability for various cluster types. In all these cases, the stable clusters have filled shells for spin up and down manifolds and consequently have no net spin magnetic moment. As mentioned above, a fundamental question is if the stability seen in filled shells can be extended to subshells. More importantly, starting from a set of degenerate electronic levels in a shell and an electron count that would fill either a majority or a minority shell, does the cluster favor a subshell filling (Hund’s rule) or break the degeneracy through structural deformation (Jahn–Teller distortion) to fill the electronic levels with a pair of electrons. As we will show, despite the formation of superatomic shells resembling those in atoms, the filling of superatomic shells does not always follow Hund’s like filling. The reason for this departure is that the electronic orbitals in superatoms, while resembling those in real atoms in shape are spread over multiple atoms. This affects the way in which the electrons fill the shells due to two competing effects. The first refers to Hund’s rule favoring high spin states in symmetric clusters as in case of atoms, a filling favored by exchange interactions. Indeed, as we will show, higher spin states can be stabilized under some conditions. However, as opposed to atoms, the clusters can undergo structural deformations that can break the orbital degeneracy and lower the energy via Jahn–Teller effect. In small metal clusters, where the structural deformations can be energetically favorable, the filling of electronic shells favors filling of electronic states with paired electrons. In the following, we revisit these competing effects by examining a cluster of 4 Au atoms.

We begin by considering the ground state of an Au_4_ cluster.^42,43^ The ground state search started with a three-dimensional structure shown in Figure 1. For each dihedral angle φ, all the sides were optimized without any symmetry constraint. The search covered both the singlet and triplet multiplicities. For each configuration, the binding energy was calculated using the following equation

$$\mathrm{BE}=4E\left(\mathrm{Au}\right)-E\left({\mathrm{Au}}_{4}\right)$$

where *E*(Au) is the energy of a single atom and *E*(Au_4_) is the energy of the cluster. Figure 1 shows the binding energy as a function of the dihedral angle. For a symmetric tetrahedral arrangement, the lowest energy state is a spin triplet. However, the energies of the singlet and triplet states decrease in energy as the structure becomes more planar and the lowest energy configuration corresponds to a planar structure. It is interesting that a planar configuration could be reconciled within a CNFEG picture. As stated earlier, for a noninteracting electron gas confined to a uniform spherical background, the electronic states order as 1S, 1P, 1D, 2S··· where the orbitals are spread over multiple atoms. As each Au atom contributes one valence electron, the ground state corresponds to an electronic configuration 1S^2^ 1P^2^. The two p-electrons could occupy P_*x*_ and/or P_*y*_ states depending on the multiplicity thus suggesting a planar configuration as the P_*z*_ orbital is unoccupied.

**Figure 1:**
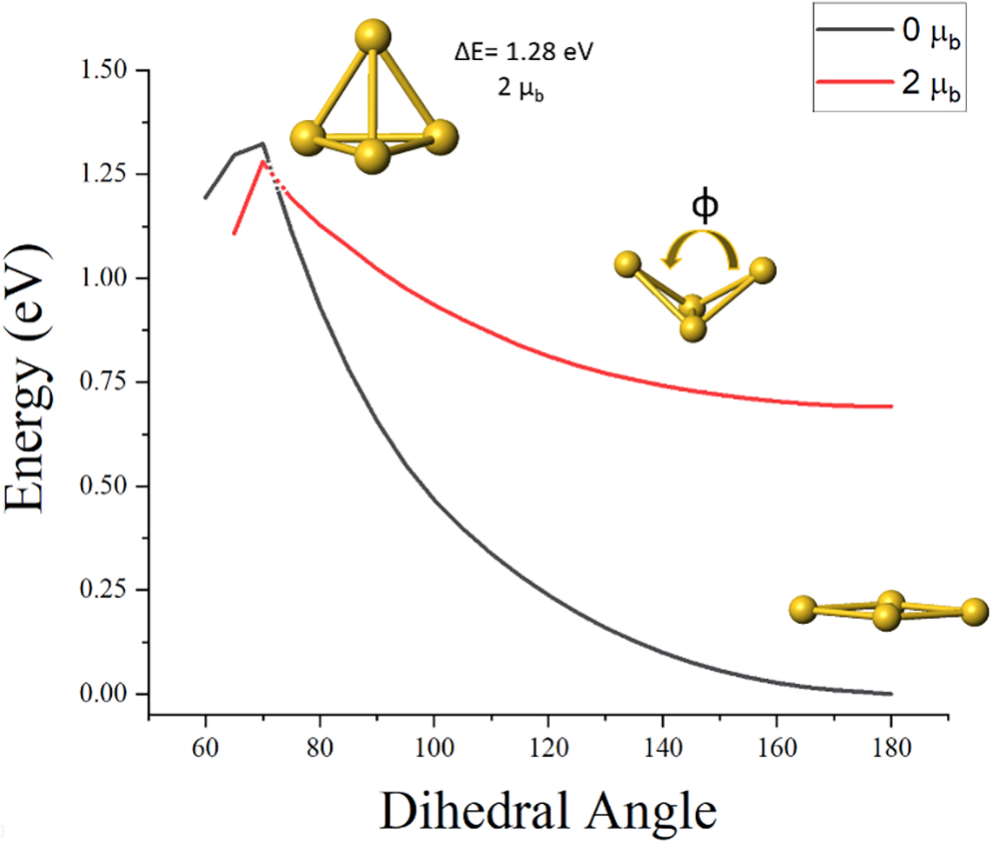
Binding Energy of the singlet and triplet configurations as a function of the dihedral angle.

For planar configurations, it is instructive to define an “order parameter”. Figure 2 shows the rhombus structure where we used the angle θ as an order parameter. An order parameter of 90° then corresponds to a square, D_4h_, structure with degenerate state corresponding to a superposition of superatomic P_*x*_ and P_*y*_ states. The lowest energy configurations can correspond to 1S^2^ 1P_*x*_^2^ or 1S^2^ 1P_*x*_^1^ 1P_*y*_^1^ occupations corresponding to singlet or triplet state. Figure 2 shows the BE as a function of θ for these singlet and triplet states. Note that the ground state corresponds to a rhombus singlet configuration with an order parameter of 60°. This spatial symmetry breaking is generally understood as a Jahn–Teller distortion where a system with partially filled degenerate states lowers its energy by undergoing a structural distortion that lowers the overall energy of the species. Note that the gain in energy due to Jahn–Teller distortion is 0.68 eV compared to the lowest energy triplet state. In fact, the energy of the singlet configuration shows large variation as a function of θ. On the other hand, the triplet configuration is largely flat with a lowest energy structure with an angle close to 72°.

**Figure 2:**
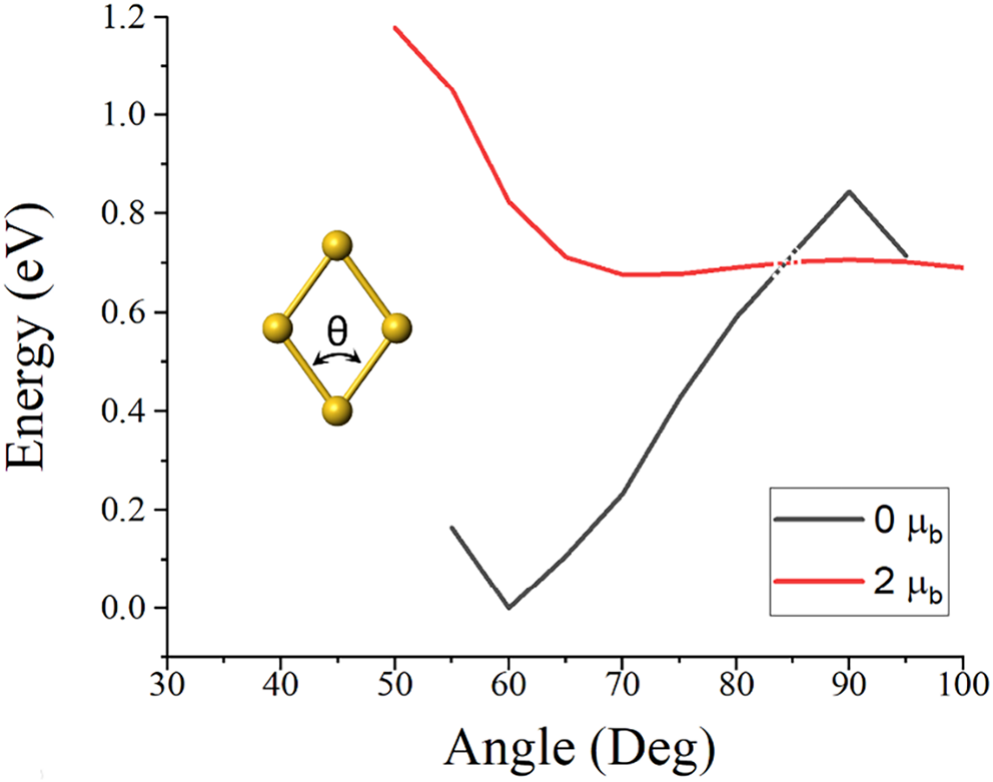
Variation of energy as a function of θ for the singlet and triplet multiplicity.

We first consider the singlet ground state. As shown in Figure 3, atoms 1 and 3 are farther than the atoms 2 and 4 indicating a bonding orbital between sites 2 and 4. To further illustrate the nature of the bond, we show in Figure 4, the molecular orbital corresponding to the singlet ground state. Note that the lowest molecular orbital is a symmetric 1S state while the highest occupied molecular orbital (HOMO) is a P_*x*_ orbital with two electrons. The occupation of the P_*x*_ orbital clearly leads to a spatial asymmetry. To further examine the result of this asymmetry, we conducted a Mulliken population analysis of the charge around each site. We found that there is a charge transfer from sites 1 and 3 to sites 2 and 4 resulting in a net quadrupole moment for the whole system. The formation of two dipoles forming the quadrupole is interesting considering the fact that the cluster consists of four identical Au atoms.

**Figure 3:**
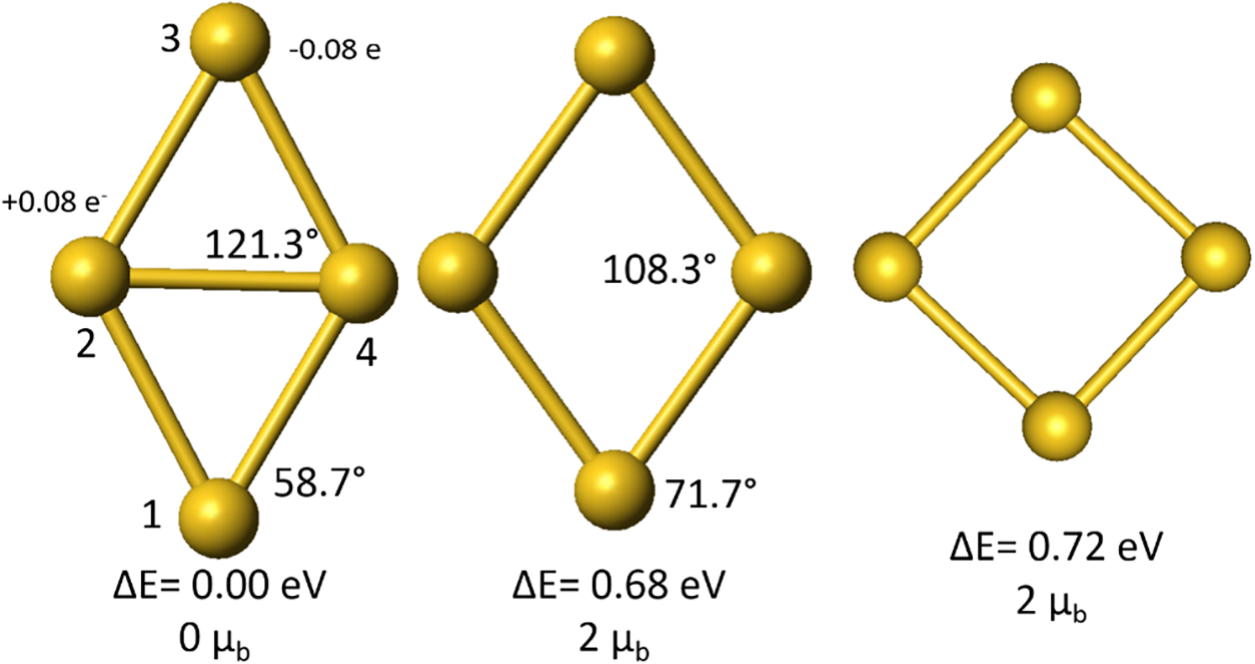
Relative energies for the singlet and triplet ground states and the triplet state with a D_4h_ symmetry.

**Figure 4:**
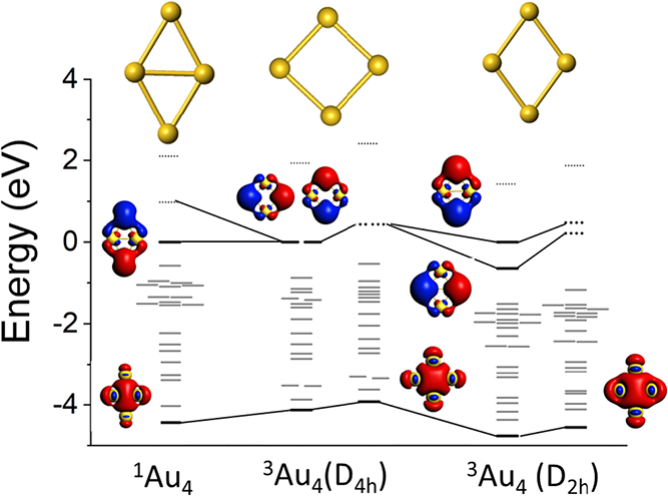
Molecular orbital corresponding to the singlet ground state, a D_4h_ square configuration and the triplet ground state.

We asked ourselves what happens if one tries to restore the spatial symmetry. This could be accomplished by going toward configurations close to 90°. Figure 3 shows that the ground state now corresponds to triplet state. Figure 4 shows that the molecular orbitals correspond to a degenerate P_*x*_ and P_*y*_ orbitals favoring a 1S^2^ 1P_*x*_^1^ 1P_*y*_^1^ configuration with spins aligned in P states in accordance with Hund’s rules. In fact, as shown in Figure 2, the singlet state reaches the highest energy at an angle of 90° indicating an instability of the spatially symmetric state. At this configuration, the triplet state is less stable than the singlet state by almost 0.72 eV. In fact, the triplet state is favored for order parameters 85° < θ < 95°. In the triplet state, all sites have same similar spin densities namely that spatial symmetry is restored. It is interesting to note that the triplet energy surface is fairly flat. This is expected as the P_*x*_ and P_*y*_ orbitals are fairly diffuse and their energy is not very sensitive to the spatial asymmetry.

To summarize, we have shown how starting from four symmetric Au atoms, the low energy can be achieved by a competition between two solutions that either break the spatial symmetry or the spin symmetry. The ground state is a rhombus arrangement that is singlet but higher spin configurations can be found by going to more symmetric atomic configurations. We now investigate how majority and minority subspaces achieve stability when both are filled with electrons.

## IV Stability due to Double Single-Shell Closures: Copper and Silver Clusters Reveal Filled Split Shells or Broken Spatial Symmetry

In the above discussion, we have outlined how nonmagnetic ground states emerge as a cluster lowers its energy by undergoing Jahn–Teller distortions to break the degeneracy of electronic states filling the HOMO with paired electrons and stabilizing the cluster with large HOMO–LUMO gap. We now consider a different scenario by considering clusters with electron count that can fill two different subshells. One of the fundamental questions is if the partial filling of a shell with unpaired electrons and broken spatial symmetry is more favorable than two filled subshells belonging to different quantum numbers? We again consider a CNFEG gas where the electronic states order as 1S, 1P, 1D, 2S, 1F, 2P··· corresponding to stability at electronic counts of 2, 8, 18, 20, 34, 40··· electrons.

To illustrate this comparison, we begin with a Cu_13_/Ag_13_ clusters where Cu and Ag atoms have filled 3*d*/4d shells and one electron in 4*s*/5s state. The clusters have 13 valence electrons derived from the 4*s*/5s atomic states.^44–47^ Now consider a situation where the majority electrons fill half the shell for 18 electron magic count (requiring 9 spin up electrons) while the minority shell correspond to half the shell originating from the magic count of 8 electrons (4 spin down electrons). In this scenario, while both the majority and minority shells are filled, they correspond to different magic numbers. Do such clusters exhibit enhanced stability under such situations.

To consider this possibility, we carried our first principles electronic structure calculations on Cu_13_ and Ag_13_ clusters. In each case, we examined the binding energy for various multiplicities to examine if the filling of subshells does result in enhanced stability. As markers of stability, we examined several parameters similar to the criterion used for identifying magic species in CNFEG. The first parameter was

$$\Delta_{2}\left(E\right)=E_{B}\left(M+1\right)+E_{B}\left(M-1\right)-2E_{B}\left(M\right)$$

where *E*_B_ is the atomization energy for a given multiplicity *M* (*E*_B_ (*M*) = (*E* (Cu_13_) – 13 *E*(Cu)); here *E* is the total energy for the cluster or atom). We also examined the HOMO–LUMO gap as a large gap points to electronic stability in that the cluster is resistant to gaining or losing an electron. We also calculated the ionization energy (I.E.) representing the energy required to remove an electron, and electron affinity (E.A.) representing the energy gained in adding an electron. The filled electronic shells generally correspond to more symmetric shapes. To this end, we examined the ellipsoidal deformation parameter defined as a prolate deformation coefficient, ε. The deformation coefficient is calculated using eq 1.^47^

1 $$\epsilon =\frac{Q_{x}+Q_{y}}{{2Q}_{z}}$$

where *Q*_*x*_, *Q*_*y*_, and *Q*_*z*_ are eigenvalues of deformation tensor in which, I runs over every ion, and *R*_I_ *_i_* is the *i*th coordinate of ion I, and *R*_I*j*_ is the *j*th coordinate of ion I relative to the center of mass. This produces a 3 × 3 matrix, and the eigenvalues are ordered so that *Q*_*z*_ > *Q*_*y*_ > *Q*_*x*_.

2 $$Q_{\mathrm{ij}}=\sum_{I}R_{Ii}R_{Ij}$$

A roughly spherical cluster will have *Q*_*z*_ ≈ *Q*_*y*_ ≈ *Q*_*x*_ and ε = 1. A prolate structure will have *Q*_*z*_ > *Q*_*y*_ ≈ *Q*_*x*_, and the prolate distortion becomes completely planar for ε = 0.

Figure 5 shows the ground state structures of Cu_13_ and Ag_13_ clusters for various multiplicities. Note that the ground state in both cases corresponds to a deformed structure as 13 electrons do not correspond to a filled shell of paired electrons and the cluster undergoes distortions to break the degeneracy as in Jahn–Teller distortion. We now examine any stability arising from subshell occupation. The results are shown in Figure 6. Note that Δ_2_ (*E*) exhibits a local enhancement in stability compared to neighboring multiplicities at a multiplicity of 6 corresponding to a majority filled subshell of 9 electrons and a minority shell filled with 4 electrons. Additionally, the HOMO–LUMO gap exhibits a local maximum, the I.E. shows a local maxima, and the E.A. shows a minimum. All these are signatures of enhanced local stability reminiscent of filled shells. It is important to highlight that as in the case of Au_4_, the local maxima at the double shell filling in Copper and Silver is less stable than the highly distorted Jahn–Teller structure. The Jahn–Teller structure is 0.76 and 0.90 eV more stable than the high spin double filled shell structure. This distortion is clearly seen in the deformation parameter that approaches 1.0 for double filled shells indicative of a minimal geometrical distortion.

**Figure 5:**
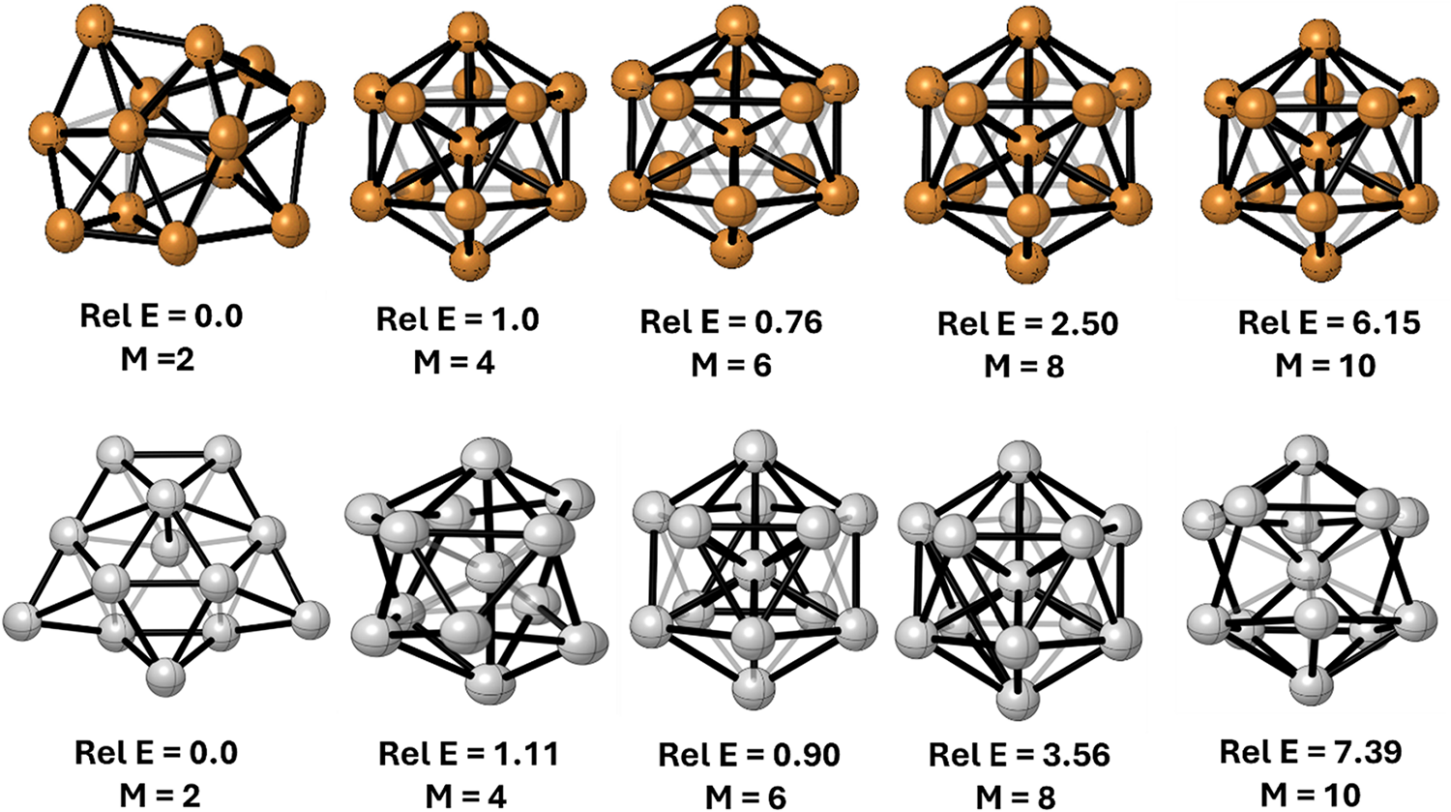
Ground state structures of Cu_13_ (top) and Ag_13_ (down) clusters for different multiplicities and relative energies.

**Figure 6:**
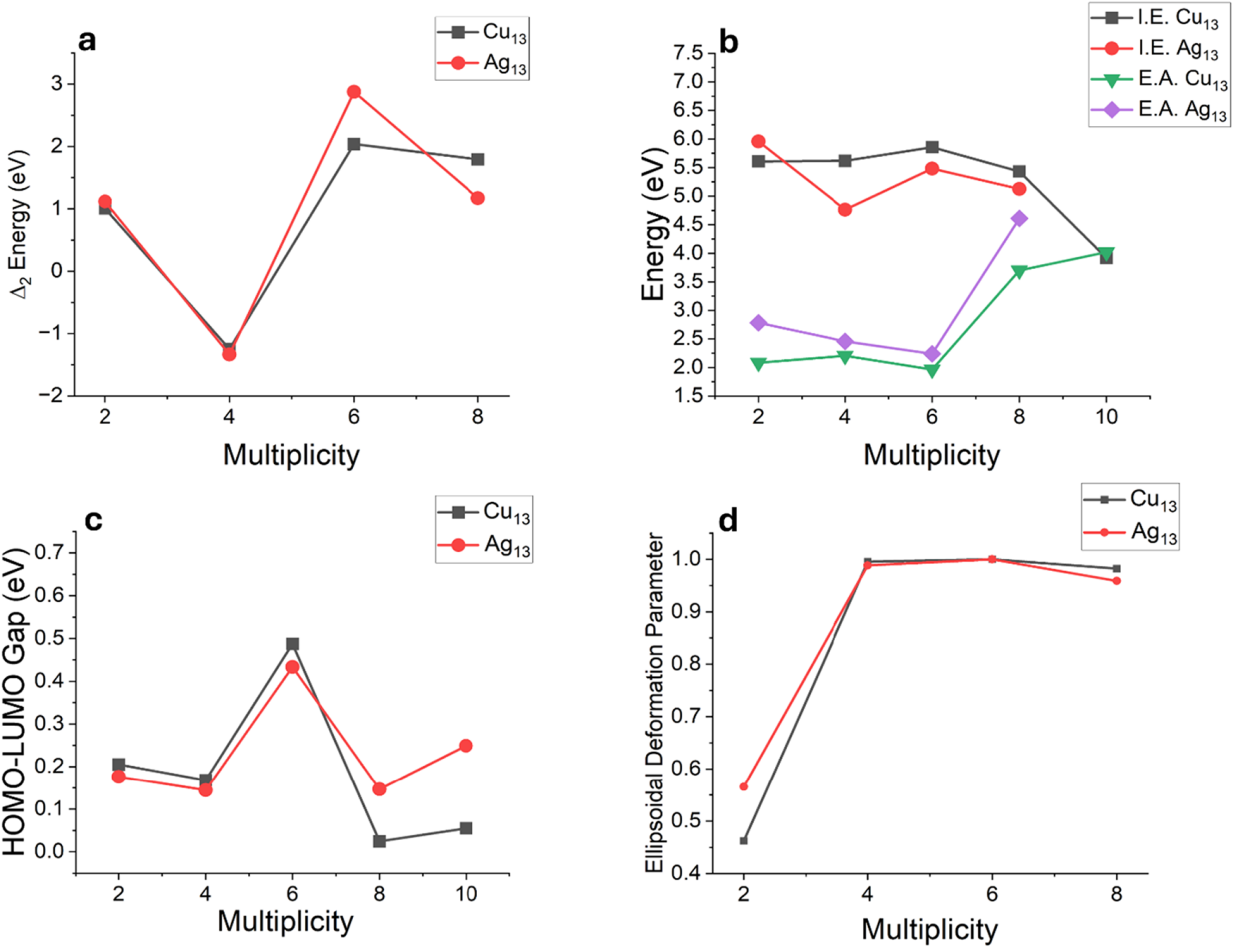
Various properties of Cu_13_ and Ag_13_ clusters as a function of the spin multiplicities: (a) Δ_2_ (*E*), (b) I.E and E.A, (c) HOMO–LUMO gap, and (d) Ellipsoidal deformation parameter.

To further demonstrate the partial shell filling, we examined the molecular orbitals in the clusters. These are shown in Figures 7 and 8. Note that the majority shell has filled 1S, 1P, and 1D orbitals while the minority shell has 1S, and 1P orbital confirming the above picture.

**Figure 7:**
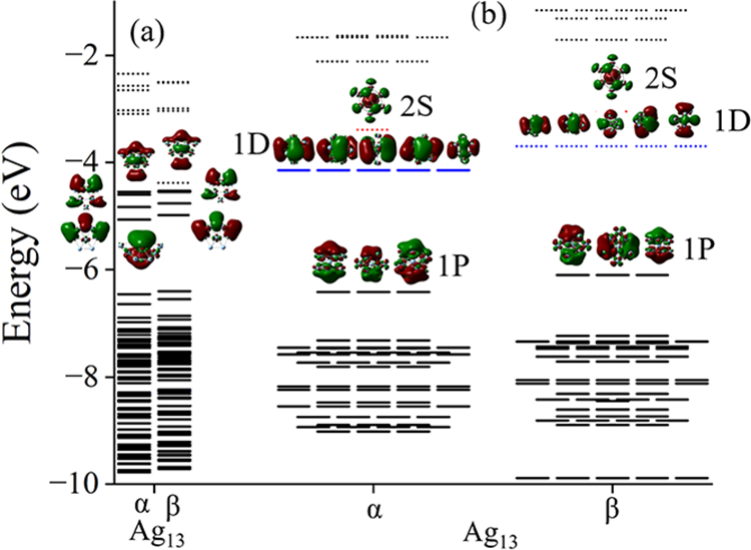
Molecular orbitals of Ag_13_. (a) ground state with doublet state and Ag_13_ (b) icosahedral geometry with sextet state. Continuous lines represent the filled states while the dotted lines represent unfilled states.

**Figure 8:**
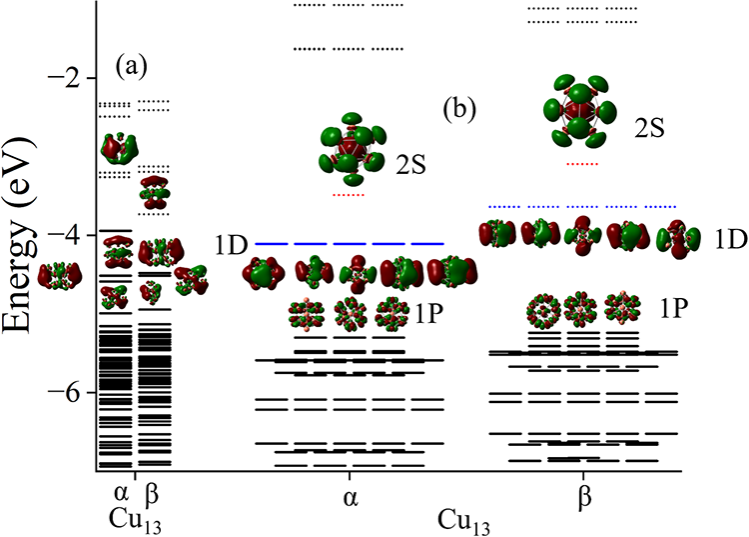
Molecular orbitals of Cu_13_. (a) Ground state with doublet state and Cu_13_ (b) icosahedral geometry with sextet state. Continuous lines represent the filled states while the dotted lines represent unfilled states.

As discussed above, while the filling of double shells leads to local stability compared to neighboring multiplicities, the ground state still corresponds to a Jahn–Teller distorted structure. Thus, the only way to have a ground state stabilized by double shell filling is to reduce structural distortions, for example, by having clusters with covalent bonds that reduce geometrical distortions. In the following, we show how it is indeed possible to form such magnetic species.

## V Stable Clusters with Two Filled subshells

In the above we have demonstrated that while the filling of subshell/subshells does enhance stability, Jahn–Teller distortions lead to more stable species with shells filled with pairs of electrons. This is because, the clusters are relatively soft and the structural distortions do not require large energy. The key to stabilize clusters with double filled shells is then to go to clusters bound by covalent bonds that stabilize symmetric structures. To this end, we examined the metal chalcogenide clusters that are marked by covalent bonds favoring octahedral structures. In these clusters, the covalent bonding leads to highly symmetric structures not amenable to structural distortions as shown by previous experimental studies and theoretical works. As discussed in section 1, the high symmetry leads to significant degeneracy in their electronic states resulting in high electronic stability for certain electron counts. In particular, as mentioned earlier, studies on various combination of octahedral TM_6_E_8_L_6_ (TM: transition metal, E: Chalcogen atoms, L: Ligand) clusters indicated that clusters with electronic counts of 96, 100, and 114 are preferentially stable.

In order to examine the stability of stable clusters with double filled shells, we first briefly review a Fe based cluster from our earlier studies. Consider the typical diamagnetic closed shell clusters Co_6_S_8_(CO)_6_ and [Re_6_S_8_(CO)_6_]^2+^. Co_6_S_8_(CO)_6_ has 114 valence electrons (6 × 9 electrons derived from Co, 8 × 6 electrons from S, and 6 × 2 electrons in the Co–CO bonds), and has a large HOMO–LUMO gap of 1.71 eV.^48–52^ Note that in this valence electron count, we did not include the electrons in the ligand itself. [Re_6_E_8_(CO)_6_]^2+^, on the other hand, has an electron count of 100 and a HOMO–LUMO gap of 1.88 eV. When compared to [Re_6_E_8_(CO)_6_]^2+^, the additional 14 electrons of Co_6_S_8_(CO)_6_ occupy A_2g_, T_1g_, and T_2u_ orbitals; these close-lying sets of high symmetry orbitals are the reason why certain electron counts are favored. To design an electronically stable dual subshell cluster that also has a net moment, we had considered a cluster [Fe_6_S_8_(CN)_6_]^5–^ that has an ideal electron count of 107 electrons (see Figure 9).^53^ We found that as opposed to clusters with 114 and 100 electrons that have singlet ground states, the ground state of the cluster has 7 unpaired electrons. The majority-filled subshell consists of orbitals corresponding to the magic 114 electron configuration (spin up channel), with 57 electrons before a large energy gap. The minority subshell configuration (spin down channel) consists of orbitals derived from the 100 electron magic configuration, with 50 electrons before its energy gap. The result is a cluster with a sizable HOMO–LUMO gap and a large spin magnetic moment resulting from 7 excess (unpaired) electrons in the majority spin channel (shown in red in Figure 9). The theoretical results were verified by earlier experiments.^53^

**Figure 9:**
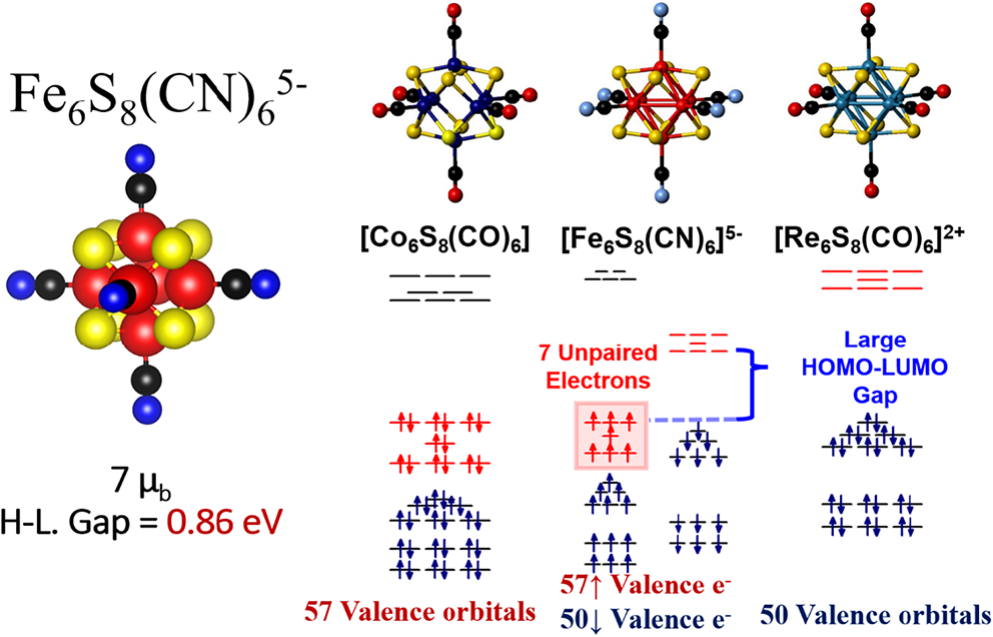
Electronic structure of Co_6_S_8_(CO)_6_, [Fe_6_S_8_(CN)_6_]^5–^, and [Re_6_S_8_(CO)_6_]^2+^. The structure, spin magnetic moment, and HOMO–LUMO gap for [Fe_6_S_8_(CN)_6_]^5–^ are shown. Valence electrons for the cluster core are assigned by counting all valence electrons from the transition metals and Sulfur, and 2 valence electrons per CO or CN^–^ molecule.

The key issue is if the electron count of 107 is unique to [Fe_6_S_8_(CN)_6_]^5–^ or other clusters also share the double shell configuration with net spin moment. This is important to establish more generally the stability of double shell fillings by considering variations in stability as a function of electron counts. We therefore considered a neutral species Fe_5_MnS_8_(CO)_6_ that also has 107 valence electrons. We also subsequently replaced a pair of Fe sites with MnCo having the same number of valence electrons leading to Mn_2_Fe_3_CoS_8_(CO)_6_ and Mn_3_FeCo_2_S_8_(CO)_6_ clusters that all have 107 electrons. All the theoretical studies were carried out without any symmetry constraint allowing for geometrical distortions. Note that the replacement of a pair of Fe sites with MnCo, while retaining the same valence count, does introduce broadening of energy levels reducing the degeneracy seen in octahedral symmetry. Our studies therefore also address how a loss of degeneracy affects the double shell stability. To examine the stability of the magnetic state, we also calculated Δ*E*_M_ which is the energy difference between next lower spin state, M, and the 7 μ_b_ state. We also examined the HOMO–LUMO gap. Figure 10 shows the ground state structures, the Δ*E*_M_, the spin magnetic moment of the ground state for the three clusters.

**Figure 10:**
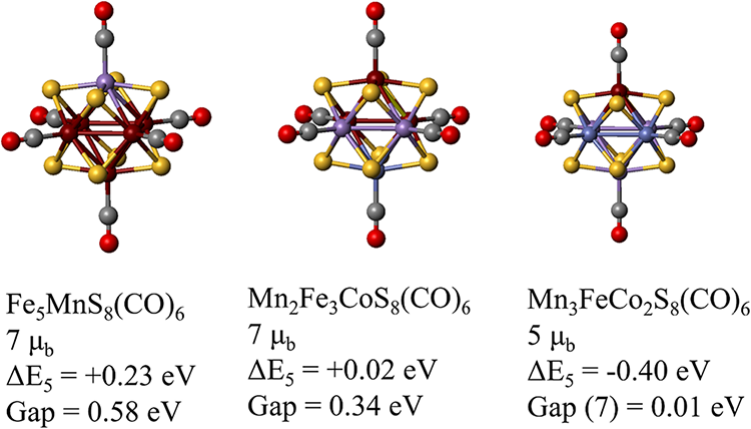
Structure, of 3 clusters with 107 valence electrons, their lowest energy spin magnetic moment, the Δ*E*_M_ which is the energy difference between next lower spin state, M, and the 7 μ_b_ state. The HOMO–LUMO gaps of the clusters are also given, and for the Mn_3_FeCo_2_S_8_(CO)_6_ cluster the gap listed is for the 7 u_b_ state.

Note that Fe_5_MnS_8_(CO)_6_ has a spin magnetic moment of 7 μ_b_ consistent with the filling of subshells as in a [Fe_6_S_8_(CN)_6_]^5–^ cluster. The cluster has a HOMO–LUMO gap of 0.58 eV and a Δ*E*_M_ of 0.23 eV. Replacing a pair of Fe sites with MnCo pair to create Mn_2_Fe_3_CoS_8_(CO)_6_, while maintaining the electron count, does decrease the symmetry of the cluster. Our studies indicate that the cluster still favors a double shell filling keeping a net spin magnetic moment of 7 μ_b_. Δ*E*_M_, however becomes almost zero. This indicates that any further reduction in symmetry by replacing a pair of Fe sites with MnCo may not retain a double filling of subshells. Our studies on Mn_3_FeCo_2_S_8_(CO)_6_ do confirm this thinking. In fact, as Figure 10 shows, the cluster now has open majority and minority shells with a net spin moment of 5 μ_b_.

We next examine the role of electron count on the dual shell filling keeping the metallic core intact. To this end, we replaced some of the CO ligands by CN ligands. Our studies focused on three clusters namely Fe_6_S_8_(CO)_4_(CN)_2_, Fe_6_S_8_(CO)_5_(CN), and Fe_6_S_8_(CO)_6_ clusters with 106, 107, and 108 electrons. Our choice was motivated by the fact that replacing CO ligands by CN ligands does not change the metallic core but only affects the overall number of valence electrons. Figure 11 shows the ground state of the clusters. Note that the clusters with 106 electrons and 108 electrons have a net spin moment of 6 μ_b_, while the cluster with 107 electrons has double filled shells with a net moment of 7 μ_b_. The clusters with 107 electrons has the highest Δ*E*_M_ and the HOMO–LUMO gap.

**Figure 11:**
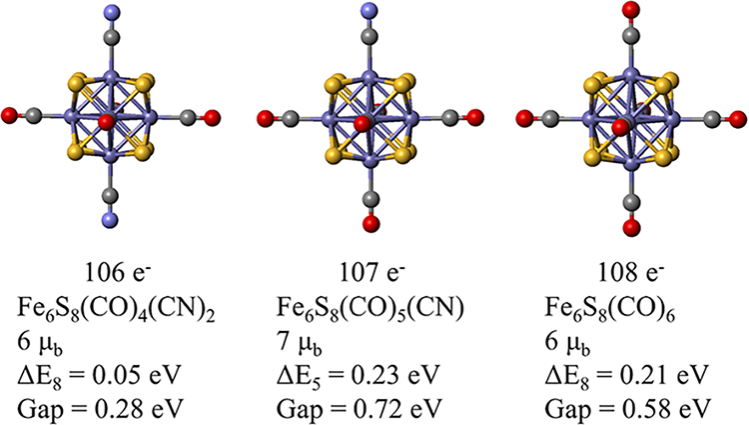
Structure of Fe_6_S_8_(CO)_4_(CN)_2_, Fe_6_S_8_(CO)_5_(CN), and Fe_6_S_8_(CO)_6_, their lowest energy spin magnetic moment, the Δ*E*_M_ which is the energy difference between next lower spin state, M, and the ground spin state, and the HOMO–LUMO gap (Gap) of the clusters.

## VI Conclusions

The present work demonstrates that the stability associated with filled shells can be extended to half-filled shells. For simple and noble metal clusters, the low energy structural deformations, generally favor clusters with shells filled with pairs of electrons even though one can see signatures of stability in symmetric clusters with filled subshells belonging to different quantum shells. The key to stabilize clusters with differential filled subshells is then to focus on clusters where the structural distortions are energetically harder and where the symmetry can retain degenerate shells. In such cases, the stable subshells belonging to different quantum states lead to energetically most stable configurations. It is in these cases that subshells filled with different quantum states lead to spin imbalance resulting in magnetic motifs. The simultaneous occurrence of energetic stability and net magnetic moments provides a unique opportunity to design stable magnetic units that are amenable to stable magnetic assemblies keeping their identity. Since the magnetic motifs are composed of multiple atoms, it will be interesting to examine the magnetic anisotropic energy and interactions between such units in cluster assembled solids.
